# Use of Humidified High Flow Nasal Oxygen in Community Palliative Care: A Case Report

**DOI:** 10.1089/pmr.2020.0026

**Published:** 2020-09-03

**Authors:** Stephen Bode, Graham Grove

**Affiliations:** Supportive and Specialist Palliative Care Service Gold Coast Hospital and Health Service, Gold Coast, Queensland, Australia.

**Keywords:** AIRVO, community palliative care, humidified high-flow nasal oxygen

## Abstract

Breathlessness is a distressing symptom that is often seen in palliative care patients with respiratory failure and it can make care in the home setting difficult. Humidified High Flow Nasal Oxygen is a relatively new intervention for respiratory failure, but it has not been researched greatly in a palliative care setting. One device with the capacity to deliver high flow humidified oxygen to spontaneously breathing patients is the myAIRVO2 humidifier.^1^ The myAIRVO2 is a humidifier with an integrated flow generator that delivers warmed and humidified respiratory gases to a spontaneously breathing patient.^1^

The following case report describes how the technology was used at home for symptom control in a 76 year old patient with severe chronic obstructive pulmonary disease with associated pulmonary hypertension. The patient was successfully discharged from hospital and managed at home using high-flow nasal oxygen for approximately one month up until his death. In this last month of life, he reported that he was more comfortable on high-flow nasal oxygen than on traditionally-administered oxygen. Humidified High Flow Nasal Oxygen is potentially beneficial to aid in symptom control for palliative care patients in an inpatient and community setting.

## Introduction

Humidified High-Flow Nasal Oxygen (HHFNOx) has been gaining in popularity and has become an important treatment in critical care in recent years.^2^ This technology is now starting to be trialed in both palliative care inpatient and community settings. One device with the capacity to deliver high flow humidified oxygen to spontaneously breathing patients is the myAIRVO2 humidifier with integrated flow generator. This system works with an air-oxygen blender allowing for a fraction of inspired oxygen (FiO_2_) of between 21% and 100%. It can generate high flows of air up to 60 L/min.^3^ The use of HHFNOx has a range of physiological benefits including pharyngeal dead space washout, reduction of nasopharyngeal resistance, positive expiratory pressure effect, alveolar recruitment, humidification, and reduction in discomfort of dyspnea.^3^ Although there is an increasing amount of experience on the use of HHFNOx in healthcare, there is very limited research on its use in a community palliative care setting.

Routine palliative care management of dyspnea involves a multidisciplinary team approach. After investigating and treating reversible causes of breathlessness, various symptomatic management strategies can be useful in improving quality of life. Patients may be provided with a breathlessness action plan and receive education on various coping strategies including the use of fans, modifying positioning for comfort, breathing exercises, and energy conservation techniques. Various medications can also relieve symptoms, including opioids and benzodiazepines.^4^ Supplemental continuous oxygen is sometimes prescribed for patients with dyspnea, primarily for those with documented hypoxemia.^5^

Patients with end stage respiratory failure, such as those with severe chronic obstructive pulmonary disease (COPD) often develop increasing oxygen requirements, ultimately requiring continuous home oxygen 24-hours a day. For patients on higher oxygen flow rates, for example, on 10-15 L/min through face mask, it can be very difficult to remove the face mask for a few minutes for everyday tasks such as eating and drinking because of severe dyspnea.

One clinical benefit of HHFNOx it that patients are typically delivered warmed high flow of oxygen through nasal cannula and this is better tolerated than traditional non-warmed oxygen.^6^ Also, because high flow rates of oxygen are delivered through nasal cannula rather than through face mask, patients are able to speak, eat and drink more easily. HHFNOx with its higher flow can improve dyspnea to a greater extent than traditional oxygen as it decreases work of breathing.^7^

When patients are discharged home from hospital they usually need to be weaned from HHFNOx back onto oxygen through traditional nasal cannula or face mask. This is especially true in districts where HHFNOx machines are not available in the community. Having an option to trial HHFNOx in the community can help facilitate home discharge for patients who are too unwell to be weaned permanently from the HHFNOx in hospital. This could potentially reduce the length of stay for some patients and allow patients to be cared for at the site of their choosing. It may help facilitate dying in the home setting rather than the hospital setting if this is in line with the patient's wishes. Using HHFNOx in the community setting may also help patients stay at home longer as there is evidence that humidification can reduce acute exacerbations of airways disease and increase time until the first exacerbation.^8^ Our case report describes one patient who trialed HHFNOx in a community setting. Written informed consent was obtained from the patient's wife after the patient died.

## Case Description

A 76 year old man with severe COPD with associated pulmonary hypertension was admitted under the respiratory physicians at the Gold Coast University Hospital after presenting with a non-productive cough and shortness of breath. The patient was referred by his respiratory physician to the palliative care team for consultation asking for advice on ongoing management of his dyspnea in the community. From the patient's perspective, shortness of breath was the main symptom concerning him. Before admission he had been on continuous home oxygen at 6L/min.The oxygen was delivered through two oxygen concentrators run in series. He did have portable oxygen bottles but no longer used them as he became house bound before his admission. His recent usual oxygen saturations (SaO_2_) were between 75 and 85%. He lived with his wife and had been essentially house bound for several months. He was able to mobilize short distances limited by his breathlessness. He had not been receiving any additional community nursing services; his wife had been assisting him with all his care needs.

His medication list was as follows: Seretide 250/ 25 mcg two puffs twice daily; tiotropium daily; clopidogrel 75 mg daily; aspirin 100 mg daily; bisoprolol 1.25 mg daily; furosemide 40 mg daily; oxycodone immediate release 5 mg two-hourly as required and atorvastatin 80 mg daily

He was reviewed by the hospital's consulting palliative care physician who recommended low dose immediate-release oral morphine as required for dyspnea and who also arranged for the patient to be linked with the Gold Coast's community palliative care team once he was discharged.

During the patient's hospital stay an MET (Medical Emergency Team) call occurred due to an episode where his SaO_2_ dropped to around 60%. This occurred after the patient had a shower and walked back to his bedside chair. He became severely breathless and anxious with an increased work of breathing. During the MET call, the patient initially responded well to increased oxygen on a non-rebreather 15 L/min mask after 20 minutes. He was then placed on HHFNOx at 50 L/min with an FiO_2_ of 50%. The respiratory team's goal over the following days was to wean the patient off HHFNOx, aiming for SaO_2_ to range between 70 and 90%. The patient used his medications as per the abfovrementioned list during this time.

For the following next few days, the patient was unable to be weaned from HHFNOx successfully as he developed increasing and intolerable breathlessness each time an attempt was made to switch the mode of oxygen delivery to a face mask. Escalating doses of immediate release opioids were unable to alleviate his symptoms adequately and side-effects precluded further uptitration. His respiratory team also felt benzodiazepines were contraindicated in the patient. The respiratory and palliative care teams discussed further options and made a decision with the patient to trial HHFNOx in the community setting as his goal was to return home. The palliative care and respiratory physiotherapists educated the patient and his wife on setup and safe use on HHFNOx.

Shortly after discharge, the patient was reviewed at home by the palliative care physiotherapist. On assessment the patient's oxygen saturation was 78% on myAIRVO2 with a flow rate of 40 L/min, FiO_2_ of 36% and the temperature set to 35°C. The patient reported that he was using HHFNOx almost continuously but that he would replace it with a Hudson face mask with 8 L/min of oxygen when he needed to mobilize to the bathroom. He reported that he felt much more comfortable when he used the HHFNOx when compared with the face mask. Although the patient did not complete any formal quality of life outcome measures he did report that he was very happy to be at home with his wife. He also reported that he particularly enjoyed that he could drink cups of tea and have good conversations with his wife when using the HHFNOx.

The patient remained at home for eight days after his discharge. Unfortunately his HHFNOx machine needed to have a filter change. As the community palliative care staff were unavailable to troubleshoot how to replace the filter for a weekend the patient needed to return to hospital as his breathlessness and anxiety increased. The next day the patient was discharged home with the HHFNOx machine having had its filter change. The patient remained at home for three weeks during which time he slowly deteriorated in terms of function. He died at home and his wife reported that he was comfortable up until his death. His wife's only negative comment about the HHFNOx was that it required daily changes of the water chamber.

## Discussion

This case demonstrates that HHFNOx can be used in a community palliative care setting to improve symptoms and quality of life and allow a patient to be cared for in the site of their choosing. In our case, HHFNOx allowed our patient to return home rather than remain in hospital for his end of life care. If HHFNOx was not available for this particular case, then the patient would have been required to remain in hospital as his symptoms were inadequately controlled to allow discharge without HHFNOx.

This case also illustrates some logistical issues with HHFNOx at home. In particular, maintenance of the machine is of key consideration. As there was no available staff member to review the machine during the weekend, our patient was required to return to hospital. Other logistical issues obviously include the availability of AIRVO or alternative HHFNOx devices in the community and the cost of purchasing or hiring these devices.

It is uncertain if the use of HHFNOx prolonged the patient's life. Without HHFNOx the severe symptoms may have necessitated higher doses of opioids and the addition of benzodiazepines and so HHFNOx did possibly allow for a longer life with less drowsiness that would otherwise have been possible. From an alternative perspective, it is possible HHFNOx prolonged the dying process for our patient. However, continued use of HHFNOx was consistent with two of the patient's goals, namely, reduced breathlessness and returning home for end-of-life care. Before commencing a trial of HHFNOx in a palliative patient it would be reasonable to require an assessment of patient function and alertness first. For example, breathless patients who are alert and still able to eat and drink may have some form of improvement in quality of life with the device. Conversely, patients who are drowsy and no longer able to eat and drink are unlikely to benefit from HHFNOx and should not be offered this therapy. There is a need for further investigation of HHFNOx in a palliative care setting to determine when it is likely to be of benefit and improve quality of life.

In our patient's case, formal outcome measures such as dyspnea scores and quality of life measures were not completed. In the future some quantitative research on the use of HHFNOx in an inpatient and community palliative care setting would be beneficial to more accurately ascertain benefits of HHFNOx for patients.

## Abbreviations Used

COPD: chronic obstructive pulmonary disease
FiO_2_: fraction of inspired oxygen
HHFNOx: humidified high-flow nasal oxygen
MET: Medical Emergency Team
